# Differential Regulation of Type I Interferon and Epidermal Growth Factor Pathways by a Human *Respirovirus* Virulence Factor

**DOI:** 10.1371/journal.ppat.1000587

**Published:** 2009-09-18

**Authors:** Grégory Caignard, Anastassia V. Komarova, Mehdi Bouraï, Thomas Mourez, Yves Jacob, Louis M. Jones, Flore Rozenberg, Astrid Vabret, François Freymuth, Frédéric Tangy, Pierre-Olivier Vidalain

**Affiliations:** 1 Laboratoire de Génomique Virale et Vaccination, Department of Virology, Institut Pasteur, CNRS URA 3015, Paris, France; 2 Laboratoire de Bactériologie-Virologie, Hôpital Lariboisière-APHP, Paris, France; 3 Unité de Génétique, Papillomavirus et Cancer Humain, Department of Virology, Institut Pasteur, Paris, France; 4 Groupe Logiciels et Banques de Données, Institut Pasteur, Paris, France; 5 Laboratoire de Virologie, Department of Virology, Hôpital Saint-Vincent de Paul, Paris, France; 6 Laboratoire de Virologie Humaine et Moléculaire, Department of Virology, CHU, Caen, France; Mount Sinai School of Medicine, United States of America

## Abstract

A number of paramyxoviruses are responsible for acute respiratory infections in children, elderly and immuno-compromised individuals, resulting in airway inflammation and exacerbation of chronic diseases like asthma. To understand the molecular pathogenesis of these infections, we searched for cellular targets of the virulence protein C of human parainfluenza virus type 3 (hPIV3-C). We found that hPIV3-C interacts directly through its C-terminal domain with STAT1 and GRB2, whereas C proteins from measles or Nipah viruses failed to do so. Binding to STAT1 explains the previously reported capacity of hPIV3-C to block type I interferon signaling, but the interaction with GRB2 was unexpected. This adaptor protein bridges Epidermal Growth Factor (EGF) receptor to MAPK/ERK pathway, a signaling cascade recently found to be involved in airway inflammatory response. We report that either hPIV3 infection or transient expression of hPIV3-C both increase cellular response to EGF, as assessed by Elk1 transactivation and phosphorylation levels of ERK1/2, 40S ribosomal subunit protein S6 and translation initiation factor 4E (eIF4E). Furthermore, inhibition of MAPK/ERK pathway with U0126 prevented viral protein expression in infected cells. Altogether, our data provide molecular basis to explain the role of hPIV3-C as a virulence factor and determinant of pathogenesis and demonstrate that *Paramyxoviridae* have evolved a single virulence factor to block type I interferon signaling and to boost simultaneous cellular response to growth factors.

## Author Summary

*Respiroviruses* are important pathogens responsible for acute respiratory tract infections associated with severe airway inflammation in children, elderly and immuno-compromised individuals. Their RNA genome encodes for structural proteins that compose viral particles, but also for virulence factors that alter cell biology to enhance virus replication and spreading. Among them, the C protein plays a critical role by blocking cellular response to type I interferons, the main antiviral cytokines secreted during virus infections. To provide molecular basis to this activity, we found that the C protein of human parainfluenza virus type 3 (hPIV3-C), the most frequent human *Respirovirus*, interacts with STAT1, a key component of type I interferon receptor complex. But hPIV3-C was also found to interact with GRB2, an adaptor molecule involved in cellular response to Epidermal Growth Factor (EGF), and to enhance cell response to this cytokine. This pathway increases protein translation, promotes cell survival and contributes to airway inflammation and mucus secretion. Thus, our findings show that hPIV3-C not only inhibits the antiviral response but also stimulates cellular response to EGF, which benefits virus replication and induces an excessive inflammation of airways during infection.

## Introduction

Viruses need to interact with host macromolecules to hijack the cellular machinery and replicate. These interactions are essential for viruses to target endocytic pathways and penetrate host cells, to recruit cellular transcription and/or translation machinery, and to achieve intracellular migration and viral particles assembly. But viruses also encode virulence factors that induce a substantial alteration of host cell functions and genetic programs to increase virus replication and spreading. For example, specific viral factors stimulate survival pathways to prevent apoptosis of infected cells or inhibit cell signaling involved in immune response. Among these pathways, IFN-α/β signaling represents a prime target for viruses because of its critical role in the induction of both innate and adaptive antiviral immune responses [1]. IFN-α/β transduce signals through direct binding to a cell surface receptor composed of two transmembrane subunits, IFNAR1 and IFNAR2c [2]. This interaction activates IFNAR1/IFNAR2c associated kinases Tyk2 and Jak1 that subsequently phosphorylate STAT2 and STAT1 transcription factors. Activated STAT1 and STAT2, altogether with IRF9, form the Interferon-Stimulated Gene Factor 3 that binds IFN-stimulated response element (ISRE) promoter sequences to induce a large antiviral gene cluster. As a consequence, most viruses that are pathogenic in vertebrates have evolved virulence factors both to block IFN-α/β expression and signal transduction downstream of IFN-α/β receptor.

Human parainfluenza virus type 1 (hPIV1) and human parainfluenza virus type 3 (hPIV3) are important human pathogens that belong to *Respirovirus* genus (*Paramyxoviridae* family; [3]). These viruses are responsible for upper respiratory tract infections and colds, but often spread to the lower respiratory tract causing bronchitis, bronchiolitis and pneumonia in young children and immuno-compromised patients. hPIV3 infection is also suspected to exacerbate chronic airway inflammatory diseases like asthma [4]. Sendai virus and bovine parainfluenza virus type 3 (bPIV3) are animal counterparts of hPIV1 and hPIV3 that infect mouse and cattle, respectively. *Respirovirus* genome is a single-strand, negative-sense RNA molecule that encodes six structural proteins (*Mononegavirales* order). While hemagglutinin-neuraminidase (HN) and fusion (F) are membrane glycoproteins associated with the envelop of hPIV3 particles, the nucleoprotein (N), the phosphoprotein (P) and the viral polymerase (L) form the ribonucleocapsid complex. The matrix protein (M) is at the interface between glycoprotein tails and ribonucleocapsids.

**Figure 1:**
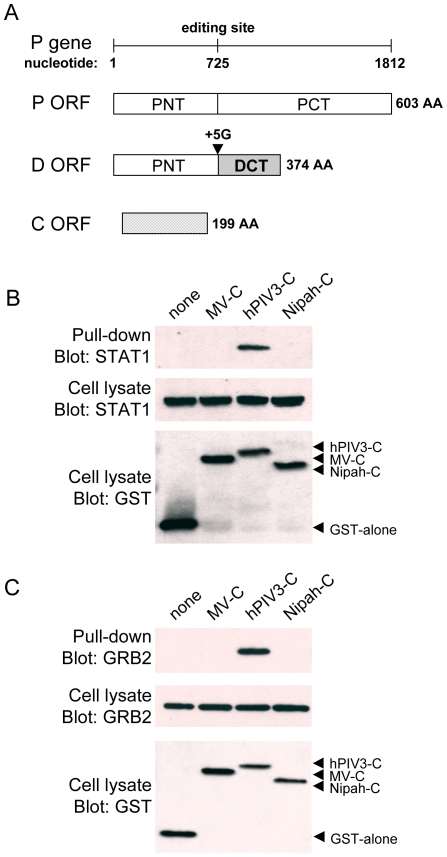
hPIV3-C interacts with endogenous STAT1 and GRB2. (A) Organization of the gene P of hPIV3 that encodes for three proteins: P, D and C. Whereas conventional transcription and translation lead to the expression of the phosphoprotein P, co-transcriptional insertion of five G residues at the editing site by the virus RNA polymerase leads to the expression of a chimeric protein called D. Insertion of one G residue can also occur during transcription but two stop codons immediately downstream of the editing site prevent the expression of the protein V that is specific of *Paramyxoviridae*. The protein C is encoded by an overlapping opened reading frame (ORF) embedded in P mRNA. (B and C) HEK-293T cells were transfected with expression vectors encoding GST alone or fused to the C proteins of measles virus (MV-C), hPIV3 (hPIV3-C), or Nipah virus (Nipah-C), and tested for the interaction with endogenous STAT1 (B) or GRB2 (C). Total cell lysates were prepared 48 h post transfection (cell lysate; middle and lower panels), and co-purifications of endogenous cellular proteins were assayed by pull-down using glutathione-sepharose beads (GST pull-down; upper panel). GST-tagged C proteins were detected by immunoblotting using anti-GST antibody, while endogenous STAT1 and GRB2 were detected with specific monoclonal antibodies.

The P gene of *Respirovirus* encodes for P but also for a panel of accessory proteins by site-specific editing of P mRNA and usage of overlapping open reading frames (ORFs). In all *Respirovirus* except hPIV1, the co-transcriptional insertion of one G residue at an editing motif midway of P mRNA leads to the expression of a chimeric protein called V. The V proteins of bPIV3 and Sendai virus bind MDA5 and suppress double-stranded RNA-stimulated IFN-β production, thereby contributing to the virus evasion of host immune response [5]. Surprisingly in hPIV3, multiple stop codons localized downstream of the editing site prevent the normal expression of a full-length V protein. As a result, P mRNA molecules edited by the addition of one G residue encode for the 242 amino acid (AA)-long N-terminal residues of P followed by only six additional AA (see Materials and Methods and [6]). But P mRNA molecules edited by the addition of five G residues encode for D, a protein exhibiting a large and specific C-terminal domain of unknown function (Figure 1A). Besides co-transcriptional edition, an overlapping ORF embedded in the first half of the P mRNA allows the expression of a single C protein (hPIV3 and bPIV3) or a nested set of four proteins called C′, C, Y1 and Y2 (Sendai virus and hPIV1). The C proteins of Sendai virus and hPIV1 have a high degree of sequence homology and have been studied in details. They are involved in the regulation of viral RNA synthesis [7],[8], the inhibition of innate immune response [9] and potentially contribute to the budding of viral particles [10]–[12]. In particular, the C protein of Sendai virus both inhibits IFN-β production [13],[14] and blocks interferon signaling downstream of IFN-α/β and IFN-γ receptors [15]–[17]. The C proteins of hPIV3 and bPIV3 only share ∼35% of sequence homology with the C proteins of Sendai virus and hPIV1, but they have also been shown to target interferon expression and signaling [5],[18]. Although expression of the C protein of hPIV3 (hPIV3-C) is essential to virulence *in vitro* and *in vivo* [19] and explains hPIV3 ability to block IFN-α/β signaling [20], host proteins that bind hPIV3-C remain unknown.

In an attempt to answer this question, we performed a yeast two-hybrid screen and we report here the identification of STAT1 and GRB2 as direct interactors of hPIV3-C. Although binding to STAT1 accounts for hPIV3-C ability to block IFN-α/β signaling, the interaction with GRB2 was unexpected. This adaptor protein bridges growth factor receptor tyrosine kinases (RTKs), like Epidermal Growth Factor (EGF) receptor, to the mitogen-activated protein kinase/extracellular signal-regulated kinase (MAPK/ERK) pathway. Upon engagement by their ligands, RTKs autophosphorylate on tyrosine residues to recruit adaptor proteins containing phosphotyrosine binding (PTB) or Src homology 2 (SH2) domains like GRB2 [21]. Once associated to RTKs by its SH2 domain, GRB2 recruits the guanine nucleotide-releasing factor son-of-sevenless (SOS) to activate Ras. Downstream events include MAPK/ERK kinase (MEK1/2) activation, which in turn phosphorylates ERK1/2. Finally, phosphorylated ERK1/2 directly or indirectly activates numerous cellular targets including transcription factors (*e.g.* Elk1, SAP1, SAP2, c-Fos, CREB, SRF) but also cellular factors that control mRNA translation like eukaryotic initiation factor 4E (eIF4E) or small ribosomal subunit S6 protein [22],[23]. Growth factor binding to RTKs regulates a multiplicity of cellular processes including proliferation, differentiation and survival. In the respiratory tract, this signaling cascade has been shown to trigger inflammation and mucus secretion by epithelial cells [24]–[27], suggesting a critical role in innate immunity [28]. However, excessive activation of this pathway could benefit to virus replication by inhibiting IFN-α/β signaling [29] and promoting infected cell survival [25]. Altogether, these data provided a rational to investigate the functional impact of hPIV3-C expression on IFN-α/β *vs* EGF receptor and MAPK/ERK signaling pathways.

## Results

### The C protein of hPIV3 interacts directly with STAT1 and GRB2

To identify cellular targets of hPIV3-C, this viral protein was used as bait in the yeast two-hybrid system to screen a human cDNA library. The screen was performed at saturation with a 10-fold coverage of the library (50.10^+6^ diploids), and positive yeast colonies growing on selective medium were analyzed by PCR and sequencing to identify binding partners of hPIV3-C. STAT1 and GRB2 were the main interactors of hPIV3-C identified in the screen with 5 and 150 yeast colonies corresponding to these cellular proteins, respectively. In both cases, cDNA clones retrieved from the screen corresponded to full-length STAT1 and GRB2 in frame with the Gal4-AD transactivation domain. To validate these interactions in human cells, GST-tagged hPIV3-C was expressed in HEK-293T cells and purified with glutathion-sepharose beads. As shown in Figure 1B and 1C, endogenous STAT1 and GRB2 co-purified with hPIV3-C. Highly divergent C proteins from measles virus (MV-C) and Nipah virus (Nipah-C) failed to do so, thereby demonstrating the specificity of identified interactions. Binding to STAT1 provides molecular basis to the inhibition of IFN-α/β signaling by hPIV3-C [18], and parallels the interaction previously identified between Sendai virus C protein and mouse STAT1 [15]. Altogether, this suggests that STAT1 is a specific cellular interactor of *Respirovirus* C proteins. In contrast, binding to GRB2 is unexpected and suggests a new function for hPIV3-C that we decided to investigate.

### hPIV3-C has opposite effects on IFN-α/β and EGF signaling pathways

**Figure 2:**
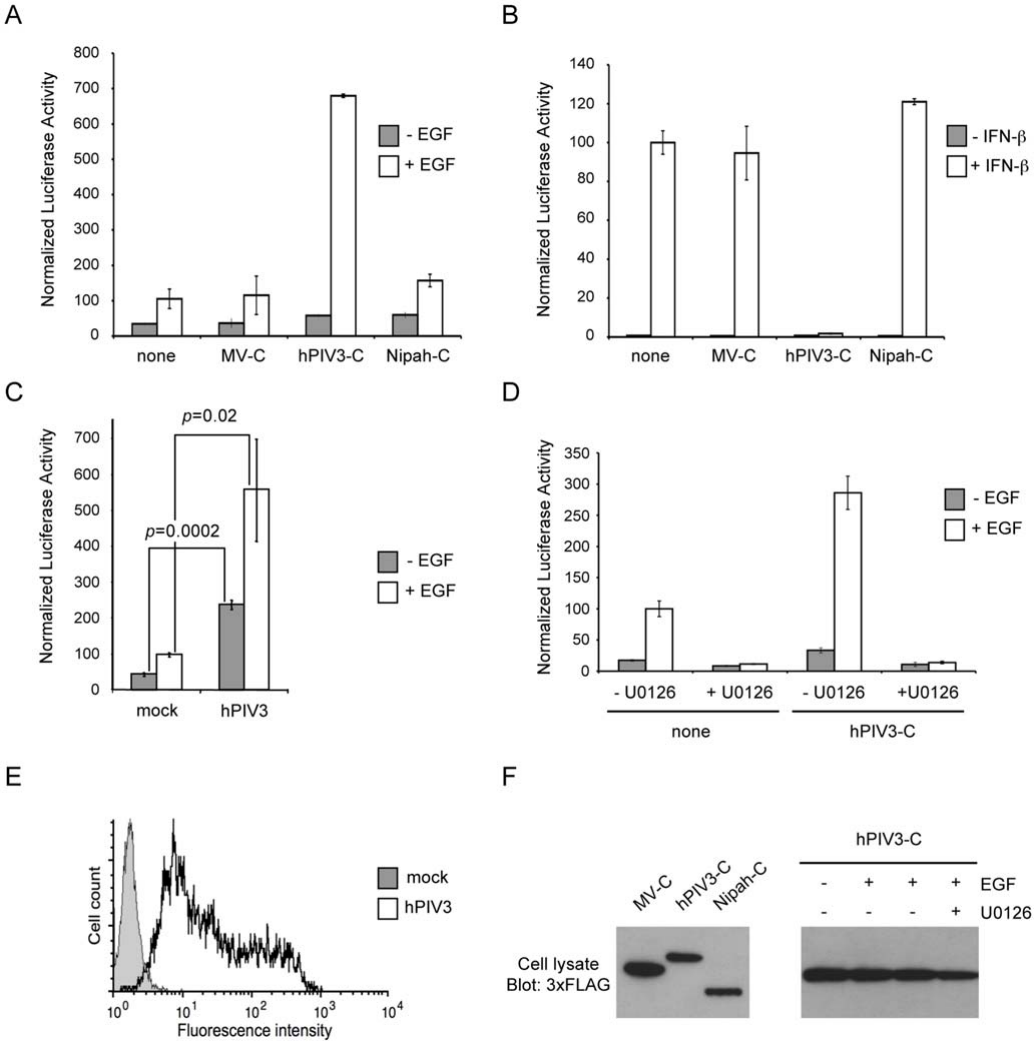
Enhanced activation of MAPK/ERK pathway and inhibition of IFN-α/β signaling by hPIV3-C. (A) HEK-293T cells were transfected with pFA2-Elk1 to express Elk1 transcription factor fused to the DNA binding domain of Gal4, pGal4-UAS-Luc that contains the firefly luciferase reporter gene downstream of a promoter sequence containing Gal4 binding site, and pRL-CMV that drives *Renilla* luciferase expression constitutively. In addition to these three plasmids, cells were co-transfected with an expression vector encoding 3×FLAG-tagged MV-C, hPIV3-C or Nipah-C or the corresponding empty vector pCI-neo-3×FLAG. 12 h after transfection, cells were starved and 6 h later EGF was added at a final concentration of 100 ng/ml. After 24 h, relative luciferase activity was determined. (B) HEK-293T cells were transfected with pISRE-Luc, a plasmid containing a luciferase gene of which expression is controlled by five ISREs, and pRL-CMV. In addition to these two plasmids, cells were co-transfected with an expression plasmid encoding 3×FLAG-tagged MV-C, hPIV3-C or Nipah-C or the corresponding empty vector pCI-neo-3×FLAG. 24 h after transfection, 1000 IU/ml of recombinant IFN-β were added. After 24 h, relative luciferase activity was determined. (C) HEK-293T cells were infected with hPIV3 (MOI = 3) and then transfected with pFA2-Elk1, pGal4-UAS-Luc, pRL-CMV vectors. 12 h later, cells were starved during 6 h and stimulated with EGF at a final concentration of 100 ng/ml. After 24 h, relative luciferase activity was determined. (D) Same experiment as (A) but 20 µM of MEK1/2 specific inhibitor U0126 was added as indicated. (A–D) All experiments were achieved in triplicate, and data represent means±SD. (E) HEK-293T cells were infected as in (C), and hPIV3-HN expression determined by immunostaining and flow cytometry analysis. (F) HEK-293T cells were transfected to express 3×FLAG-tagged MV-C, hPIV3-C or Nipah-C as described in (A) and (B), and relative expression levels were determined 36 h later by western blot analysis (left panel). In a parallel experiment, HEK-293T cells were transfected to express hPIV3-C and were cultured with or without EGF in the presence or absence of U0126 as described in (D). hPIV3-C expression level was determined by western blot analysis (right panel).

The adaptor protein GRB2 plays a critical role in coupling signal from growth factor receptors to MAPK/ERK signaling pathway. To address the question of hPIV3-C interference with this pathway, we used a trans-reporter gene assay that measures Elk1 activation by ERK1/2. In this system, Elk1 transcription factor is fused to the DNA binding domain of Gal4 (Gal4-DB) and binds the promoter sequence of a luciferase reporter gene. Upon stimulation with a growth factor like EGF, Elk1 is activated as assessed by a significant increase in luciferase expression. Surprisingly, we observed a 6-fold enhancement in this cellular response to EGF when 3×FLAG-tagged hPIV3-C was expressed in HEK-293T cells (Figure 2A). Same results were obtained when using hPIV3-C without a tag (14-fold enhancement) or tagged with the red fluorescent protein Cherry (7-fold enhancement). In contrast to hPIV3-C, neither MV-C nor Nipah-C enhanced Elk1 activity upon EGF stimulation (Figure 2A) whereas expression levels of hPIV3-C, MV-C and Nipah-C were similar in this system (Figure 2F, left panel). Elk1 activity was also enhanced by hPIV3-C expression in Vero and Hela cells as well as BEAS-2B and A549, two epithelial cell lines that originate from the respiratory tract, which is the tissue targeted by hPIV3 *in vivo* (Table 1). The effect of hPIV3-C in these different cell lines was highly significant (see *p*-values in Table 1) although relatively modest when compared to HEK-293T cells. This is probably because our reporter system requires the co-transfection of four plasmids and Vero, Hela, BEAS-2B and A549 cells are more difficult to transfect than HEK-293T. In parallel experiments, cellular response to IFN-α/β was monitored using a *cis*-reporter gene, of which expression is controlled by five ISREs. As previously reported [18], we found that hPIV3-C efficiently blocked IFN-α/β signaling (Figure 2B) as opposed to what we observed for the EGF pathway. Again, MV-C or Nipah-C was unable to do so. Altogether, these results show that hPIV3-C enhances the cellular response to EGF in addition to its ability to block IFN-α/β signaling.

**Table 1:** hPIV3-C protein expression increases EGF signaling.

	none	MV-C	hPIV3-C	Nipah-C
HEK-293T	1	1.1±0.2	**6.3**±0.2 (*p* = 0.0005)	1.4±0.2
Hela	1	1.1±0.02	**2.4**±0.07 (*p* = 3.10^−5^)	1.2±0.01
Vero	1	0.9±0.03	**2.2**±0.02 (*p* = 0.0002)	0.8±0.06
A549	1	ND	**2.2**±0.3 (*p* = 0.005)	ND
BEAS-2B	1	ND	**2.7**±0.8 (*p* = 0.02)	ND

As described in Figure 2, HEK-293T, Hela, Vero, A549 or BEAS-2B cells were transfected with pFA2-Elk1, pGal4-UAS-Luc, pRL-CMV to measure the activation level of MAPK/ERK signaling pathway. Cells were co-transfected with plasmids encoding 3×FLAG-tagged MV-C, hPIV3-C or Nipah-C or the corresponding empty vector pCI-neo-3×FLAG. 12 h after transfection, cells were starved and stimulated 6 h later with 100 ng/ml of EGF. After 24 h, expression of luciferase was quantified. Results were normalized so that reporter activity in cells transfected with a control vector equals 1. Experiments were performed in triplicates and data represent means±SD.

We also determined if similar effects on the EGF pathway were observed in infected cells. HEK-293T cells were infected with hPIV3 (MOI = 3) and then transfected with Elk1 activity reporter plasmids. Infection of HEK-293T cells was confirmed by anti-hPIV3 hemagglutinin-neuraminidase (hPIV3-HN) immunostaining and flow cytometry analysis (Figure 2E). Like hPIV3-C alone, hPIV3 infection enhanced Elk1 activity upon EGF stimulation (Figure 2C). Interestingly, hPIV3 infection induced a significant level of Elk1 activity in the absence of EGF stimulation. This suggests that in addition to hPIV3-C interaction with GRB2, other mechanisms modulate MAPK/ERK pathway during hPIV3 infection.

Finally, to demonstrate that enhancement of Elk1 activation by hPIV3-C is completely dependent on ERK1/2 activation, HEK-293T cells were pre-treated with MEK1/2 inhibitor U0126 before stimulation with EGF. This molecule targets MEK1/2 and totally abrogates downstream phosphorylation and activation of ERK1/2 [30]. As shown in Figure 2D, Elk1 activation was blocked by U0126, whereas hPIV3-C expression was maintained (Figure 2F, right panel). This demonstrates that hPIV3-C is acting through ERK1/2 stimulation. Altogether, these results support a model where hPIV3-C interaction with GRB2 enhances cellular response to growth factors as assessed by an increased activation of MAPK/ERK pathway.

### Phosphorylation of ERK1/2, eIF4E and small ribosomal subunit S6 protein are stimulated by hPIV3-C expression or hPIV3 infection

**Figure 3:**
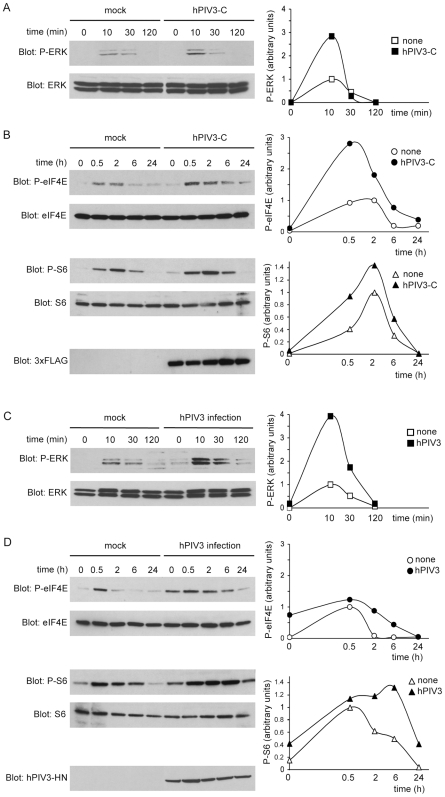
Stimulation of ERK1/2, eIF4E and S6 phosphorylations by hPIV3-C expression or hPIV3 infection. (A, B, C and D) HEK-293T cells were either transfected with an expression vector encoding 3×FLAG-tagged hPIV3-C (A, B) or infected with hPIV3 (MOI = 3) (C, D). After 24 h, cells were starved for 12 h, and then stimulated with 100 ng/ml of EGF. Phosphorylation of ERK1/2 was measured at 10 min, 30 min and 2 h (A and C) whereas phosphorylation of eIF4E on Ser209 and S6 phosphorylation on Ser235/236 were determined at 30 min, 2 h, 6 h and 24 h after EGF stimulation (B and D). Relative phosphorylation levels of ERK1/2, eIF4E and S6 were obtained by densitometric analysis of the gels (right panels). Expression of 3×FLAG-tagged hPIV3-C in transfected cells was detected by anti-3×FLAG immunoblotting (B), whereas hPIV3 infection was confirmed by anti-hPIV3 hemagglutinin-neuraminidase (hPIV3-HN) immunoblotting (D).

To further document hPIV3-C impact on MAPK/ERK signaling pathway, we compared the kinetic of ERK1/2 phosphorylation in HEK-293T cells expressing hPIV3-C or not. Cells were transfected with 3×FLAG-tagged hPIV3-C or a control plasmid and 24 h post transfection, they were starved before stimulation with EGF. ERK1/2 phosphorylation was determined at 10, 30 and 120 min after stimulation. As illustrated by one representative experiment in Figure 3A, EGF stimulation induced ERK1/2 phosphorylation in control cells but signal was markedly and reproducibly increased by hPIV3-C expression at maximum phosphorylation time point (1.4 to 2.8 fold increase; *p* = 0.005; n = 4).

We then determined the phosphorylation level of two downstream targets of this pathway that are involved in the control of mRNA translation, the translation initiation factor eIF4E and the ribosomal protein S6 (Figure 3B). Before EGF stimulation, low levels of phosphorylated eIF4E and S6 were detectable in mock-treated cells (Figure 3B and 3D). hPIV3-C expression had virtually no effects on this background. Thus, eIF4E and S6 phosphorylation levels were determined at different time-points after EGF stimulation. Because ERK1/2 activation precedes eIF4E and S6 phosphorylation, maximal phosphorylation occurs at later time points and was determined at 30 min, 2 h, 6 h and 24 h after stimulation. As observed for ERK1/2, phosphorylation levels of eIF4E and S6 were enhanced by hPIV3-C expression when stimulating the cells with EGF.

To validate these observations in infected cells, HEK-293T cells were infected with hPIV3 (MOI = 3) and 24 h later, cells were starved for 12 h before stimulation with EGF. Like hPIV3-C expression alone, hPIV3 infection enhanced ERK1/2 phosphorylation at the peak of induction, *i.e.* 10 min after adding EGF to the cells (Figure 3C). Interestingly, hPIV3 infection of A549 cells also enhanced ERK1/2 phosphorylation but the induction profile was different. Indeed, ERK1/2 phosphorylation was not significantly increased at the peak of induction, but the signal was boosted by hPIV3 infection at late time points (Figure S1). The same profile was observed when eIF4E and S6 phosphorylation levels were analyzed in infected HEK-293T cells. hPIV3 infection sustained the phosphorylation of these two translation factors at late time points, but showed no increase at the peak of stimulation, *i.e.* 30 min after adding EGF to the cells (Figure 3D). This could relate to the fact that hPIV3 infection also induces low levels of eIF4E and S6 phosphorylation in the absence of EGF stimulation (Figure 3D). This is reminiscent to what was observed for Elk1 (Figure 2C), and suggests that hPIV3 infection induces a basal activation of MAPK/ERK pathway leading to the constitutive phosphorylation of downstream targets.

**Figure 4:**
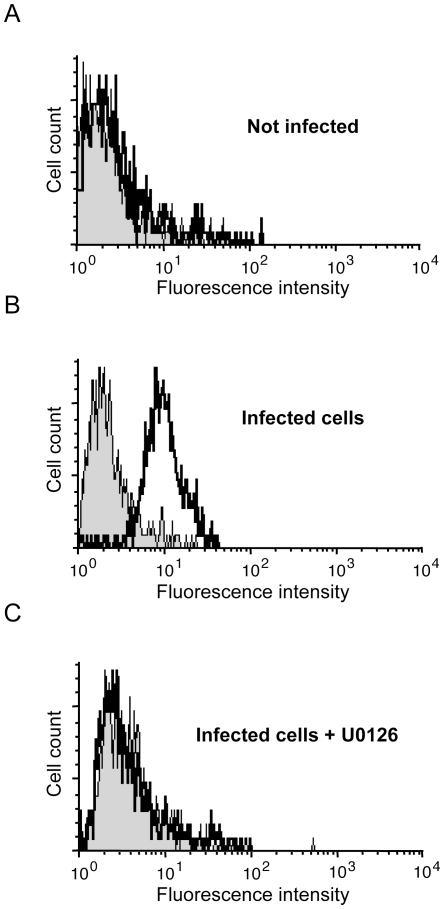
MEK1/2 inhibitor U0126 inhibits hPIV3 protein synthesis in infected cells. (A, B and C) HEK-293T cells were left untreated (A and B) or treated with 20 µM of U0126 (C) for 2 h. (B and C) cells were infected with hPIV3 (MOI = 1) and maintained in absence (B) or presence of U0126 (C). 48 h later, expression of hPIV3-HN was quantified by immunostaining and flow cytometry analysis. Isotypic control (grey shadow) and hPIV3-HN expression (thick line) are shown for each experiment.

Altogether, these data demonstrate that hPIV3 infection or hPIV3-C expression alone both enhance MAPK/ERK pathway activation in EGF-stimulated cells. Several RNA viruses require an activated MAPK/ERK pathway to produce viral components and replicate properly (for review see [31]). To test if the same was true for hPIV3, cells were treated for 2 h with MAPK/ERK pathway inhibitor U0126 and infected with hPIV3 (MOI = 1). Two days after infection, cell surface expression of hPIV3-HN was detected by immunostaining and flow cytometry. U0126 completely blocked the expression of hPIV3-HN in hPIV3-infected cells (Figure 4), whereas the same inhibitor had no effect when cells were infected with MV (Figure S2). Altogether, this demonstrates that MAPK/ERK signaling is essential for the expression of hPIV3 proteins and suggests that hPIV3 manipulates this pathway to increase replication efficiency.

### The C-terminal region of hPIV3-C binds STAT1 and GRB2

**Figure 5:**
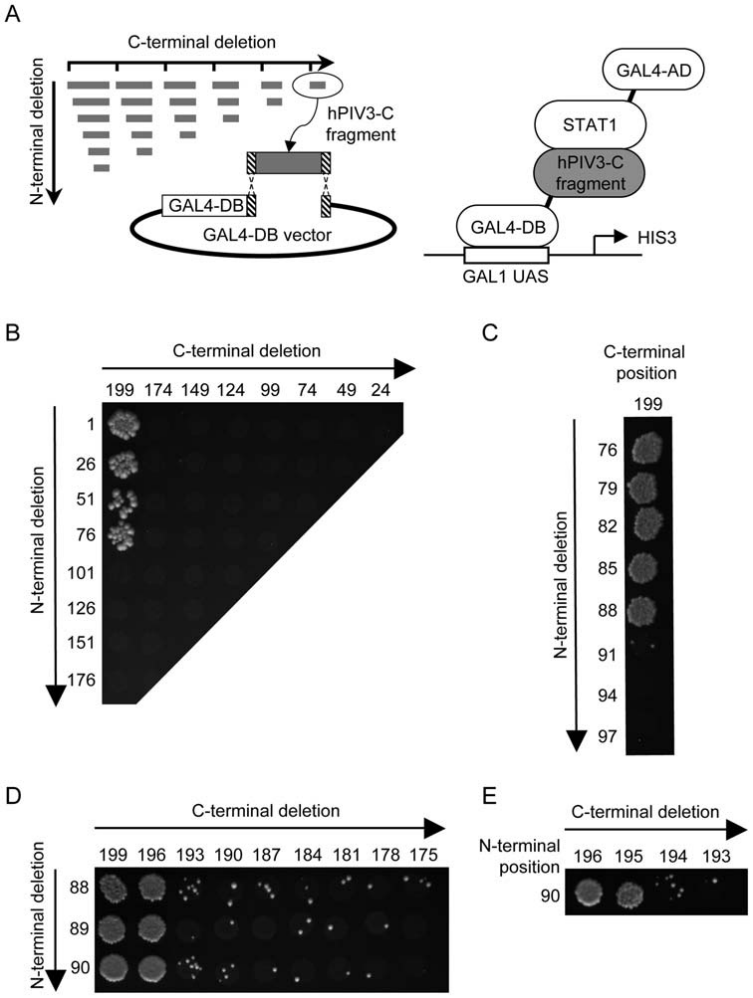
Systematic deletion-based mapping of a minimal hPIV3-C region interacting with STAT1. (A) Fragments of hPIV3-C were generated by PCR using a matrix combination of specific primers (left panel), and introduced into Gal4-DB vector by gap-repair in yeast cells expressing STAT1 fused to Gal4-AD (right panel). Yeast cells were grown on selective medium lacking histidine and supplemented with 10 mM of 3-amino-triazole (3-AT) to test the interaction-dependent transactivation of HIS3 reporter gene. Vertical and horizontal axes indicate first and last AA residues of each fragment tested, respectively. (B), (C), (D) and (E) correspond to four iterations of this process. (E) The fourth led to the identification of a 106 AA encompassing position 90 to 195 of hPIV3-C as the minimal STAT1 binding domain.

**Figure 6:**
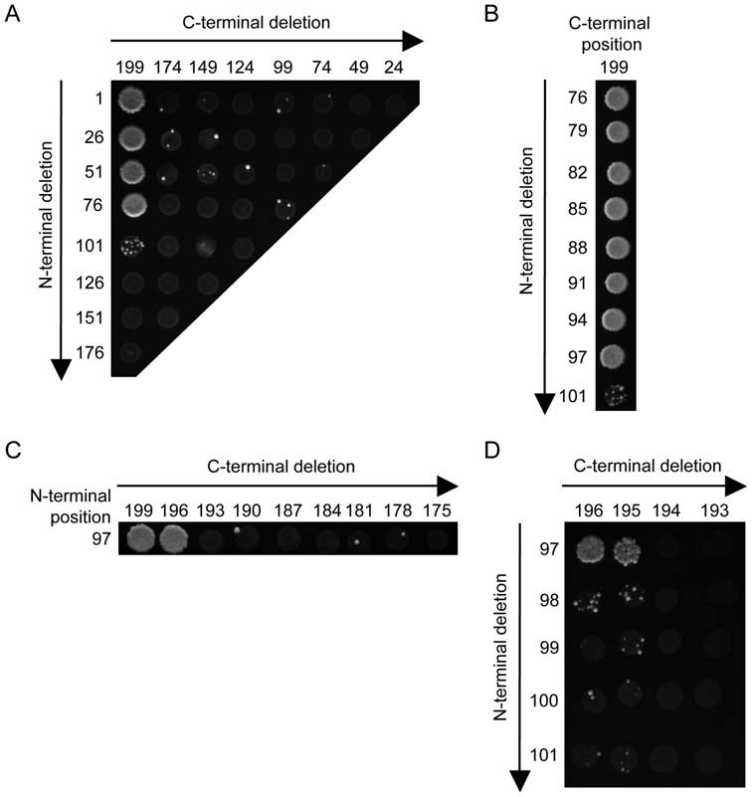
Identification of a minimal hPIV3-C region interacting with GRB2. Fragments of hPIV3-C were generated and tested for their ability to interact with GRB2 following the procedure described in Figure 5A. (A) The first iteration identified a GRB2 binding region of 124 AA. After two (B), three (C) and four additional rounds (D), this domain was finally reduced to a minimal GRB2 binding motif of 99 AA. This binding domain, encompassing position 97 to 195, is contained in the STAT1 binding region of hPIV3-C previously identified in Figure 5.

To better understand how hPIV3-C targets both the IFN-α/β and EGF signaling pathways, we characterized the functional domains of hPIV3-C that bind STAT1 and GRB2. To do so, we generated by PCR a full matrix of hPIV3-C overlapping fragments and tested their ability to interact with either STAT1 or GRB2 in the yeast two-hybrid system (Figure 5 and 6). Both forward and reverse primers were designed every 75 nucleotides along hPIV3-C sequence and fused to appropriate tails to allow gap-repair recombination with linearized Gal4-DB yeast two-hybrid vector (Figure 5A). All possible combinations of forward and reverse primers were used to amplify hPIV3-C fragments. Finally, corresponding PCR products were transformed in a yeast strain expressing Gal4-AD fused to either STAT1 or GRB2, and growth on selective medium was used to detect potential interactions. A 124 (AA)-long peptide encompassing position 76 to 199 located in the C-terminal half of hPIV3-C was sufficient to bind STAT1 (Figure 5B) or GRB2 (Figure 6A). In an iterative process, we then generated a second, a third and a fourth set of hPIV3-C fragments corresponding to one-by-one AA deletions (Figure 5C–E and Figure 6B–D), allowing to further reduce the STAT1 and GRB2 binding motifs to minimal peptides. A 106 AA peptide encompassing residues 90 to 195 of hPIV3-C was sufficient to observe the interaction with STAT1 (Figure 5E). The binding region to GRB2 was virtually the same, encompassing AA 97 to 195 (Figure 6D).

**Figure 7:**
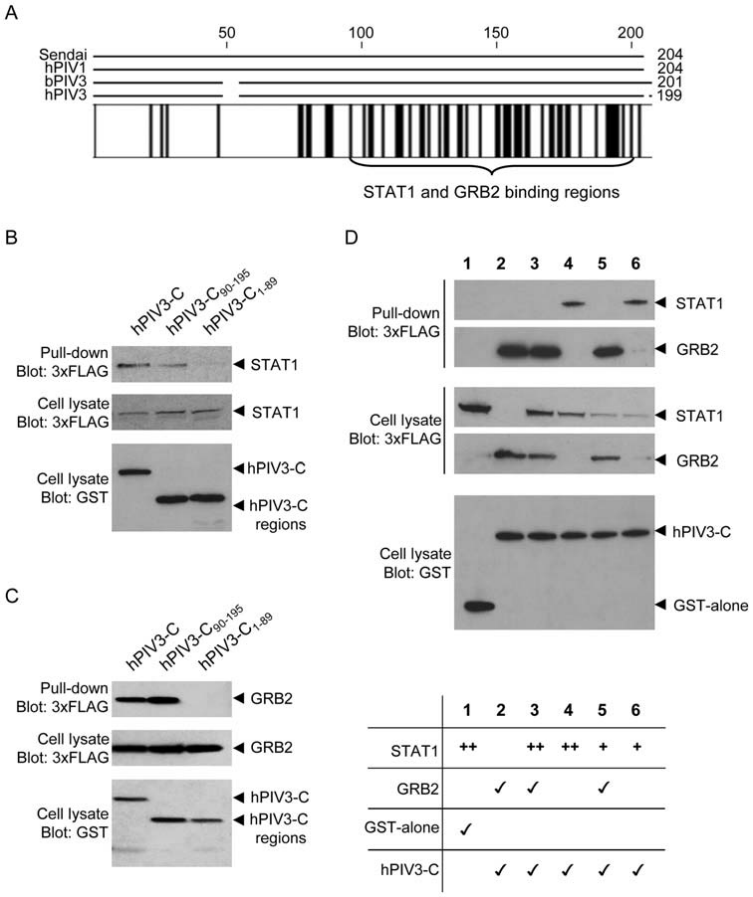
STAT1 and GRB2 bind to the same region of hPIV3-C. (A) The C proteins of *Respiroviruses* are highly conserved at their C-terminus. Sequences from Sendai virus (NC_001552), human parainfluenza virus type I (hPIV1; NC_003461), bovine parainfluenza virus type 3 (bPIV3; NC_002161) and hPIV3 (EU719627.1) were aligned with CLC Workbench 4.0.1. Identical residues are highlighted by black boxes. (B and C) Full-length hPIV3-C, N-terminal region hPIV3-C_1-89_ and C-terminal region hPIV3-C_90-195_ were tested for their ability to interact with STAT1 or GRB2. HEK-293T cells were transfected to co-express GST fused to full-length hPIV3-C or fragments and 3×FLAG-tagged STAT1 (B) or GRB2 (C). Total cell lysates were prepared 48 h post transfection (cell lysate; middle and lower panels), and co-purifications of 3×-FLAG-tagged proteins was assayed by GST pull-down (upper panel). Tagged proteins were detected by immunoblotting using anti-GST or anti-3×FLAG antibodies. (D) GRB2 and STAT1 compete for hPIV3-C binding. GST alone (line 1) or GST-tagged hPIV3-C (line 2–6; 300 ng/well in a 6-well plate) were expressed in HEK-293T cells together with 600 ng/well (line 3–4; “++”) or 150 ng/well (line 5–6; “+”) of 3×-FLAG-tagged STAT1 and/or 3×-FLAG-tagged GRB2 (line 2, 3 and 5; 600 ng/well). Total cell lysates were prepared 48 h post transfection (cell lysate; middle and lower panels), and co-purification of 3×-FLAG-tagged proteins was assayed by GST pull-down (upper panel).

The C-terminal region of hPIV3-C required to bind STAT1 and GRB2 in the yeast two-hybrid system is highly conserved among *Respiroviruses* (Figure 7A) and suspected to fold into a structured coiled-coil domain [18]. Furthermore, virtually the same C-terminal region of Sendai virus C protein (AA 85-204) was previously reported to mediate the interaction with mouse STAT1 [32]. To further validate our observations performed in the yeast two-hybrid system, we retested by co-affinity purification the ability of hPIV3-C fragment encompassing AA 90-195 (hPIV3-C_90-195_) to interact with STAT1 and GRB2 in HEK-293T cells. GST-tagged hPIV3-C_90-195_ was expressed together with 3×-FLAG-tagged STAT1 or GRB2, and purified with glutathion-sepharose beads. Full-length hPIV3-C and the N-terminal region encompassing AA 1-89 (hPIV3-C_1-89_) were used as positive and negative controls, respectively. As shown in Figure 7B and 7C, hPIV3-C_90-195_ interacted with STAT1 and GRB2, whereas hPIV3-C_1-89_ did not. Although hPIV3-C_90-195_ interacted with GRB2 as efficiently as full-length hPIV3-C (Figure 7C), interaction with STAT1 was weaker suggesting that more residues contribute to the stabilization of this interaction (Figure 7B). Altogether, these results confirm that AA 90-195 of hPIV-3 include both STAT1 and GRB2 binding sites.

Although STAT1 and GRB2 essentially bind to the same region of hPIV3-C as demonstrated above, it remained unclear whether these interactions are mutually exclusive. To answer this question, a competition experiment was designed where GST-tagged hPIV3-C was co-expressed with STAT1 in the presence or absence of GRB2 (Figure 7D). In this setting, GRB2 expression prevents STAT1 co-purification together with GST-tagged hPIV3-C. This validates our finding that STAT1 and GRB2 interact with the same region of hPIV3-C, and demonstrates that STAT1 and GRB2 compete for hPIV3-C binding. Interestingly, GRB2 interaction with hPIV3-C was not affected by STAT1 expression (Figure 7D and data not shown), suggesting that GRB2 has a higher affinity for hPIV3-C than STAT1.

### Both the N- and C-terminal domains of hPIV3-C are required for its activity

**Figure 8:**
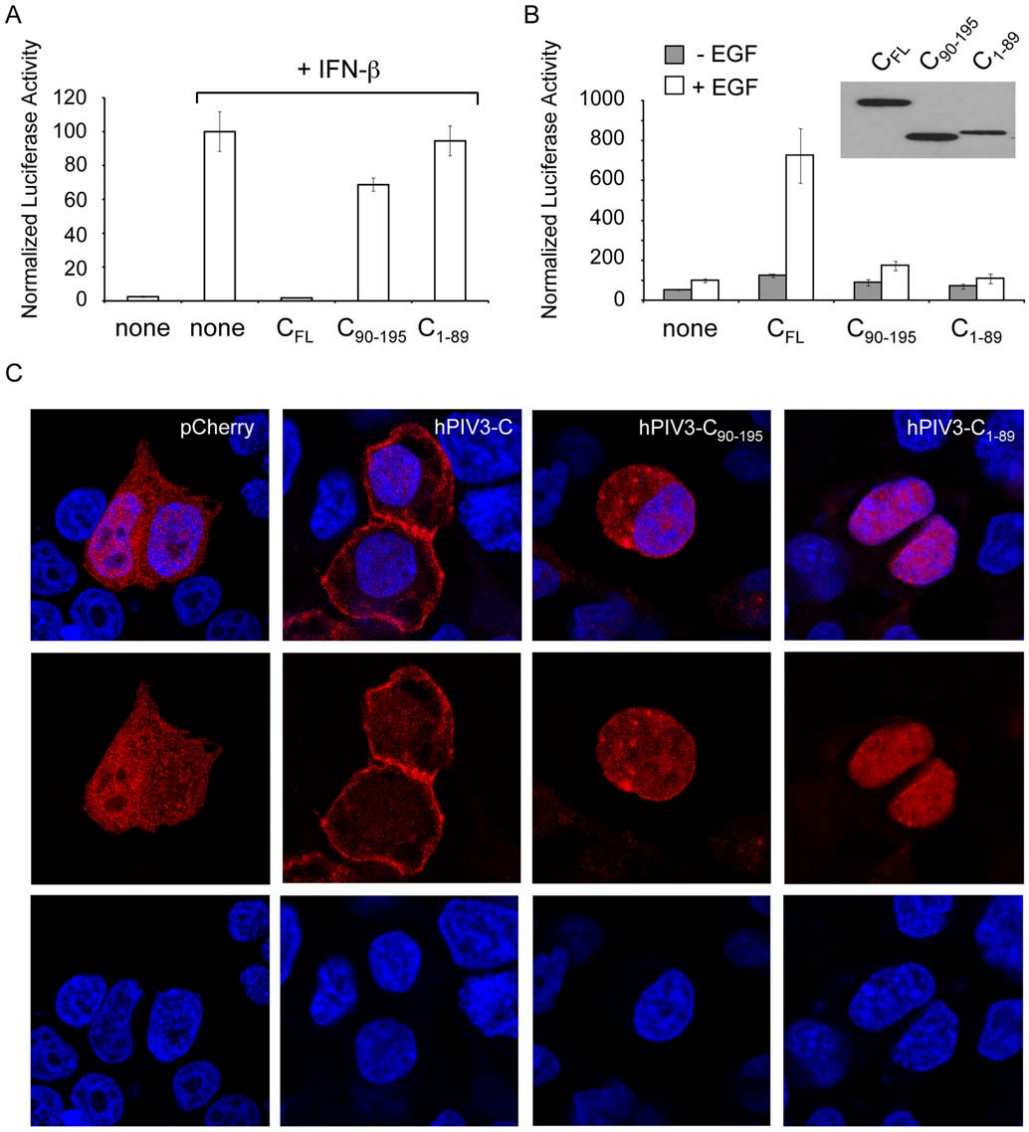
Full-length hPIV3-C is required to modulate IFN-α/β and EGF signaling. (A and B) hPIV3-C_90-195_ and hPIV3-C_1-89_ were tested for their ability to modulate IFN-α/β or EGF signaling. (A) As described in Figure 2, HEK-293T cells were transfected with pISRE-Luc and pRL-CMV to determine the activation level of IFN-α/β signaling pathway. Cells were co-transfected with expression plasmids encoding 3×FLAG-tagged full-length hPIV3-C (C_FL_) or fragments. 24 h after transfection, 1000 IU/ml of recombinant IFN-β were added. After 24 h, relative luciferase activity was determined. Experiments were performed in triplicates, and data represent means±SD. (B) As described in Figure 2, cells were transfected with pFA2-Elk1, pGal4-UAS-Luc and pRL-CMV to determine the activation level of MAPK/ERK signaling pathway. Cells were co-transfected with expression plasmids encoding full-length hPIV3-C (C_FL_) or fragments. 12 h later, cells were starved during 6 h and stimulated with EGF at a final concentration of 100 ng/ml. After 24 h, relative luciferase activity was determined. Experiments were performed in triplicate, and data represent means±SD. As a control, relative expression levels of C_FL_, C_90-195_ and C_1-89_ were determined by western blot analysis (upper right panel). (C) Full-length hPIV3-C, hPIV3-C_90-195_ or hPIV3-C_1-89_ was expressed in fusion downstream of the red fluorescent protein Cherry to determine subcellular localization in HEK-293T cells. 24 h after transfection, cells were fixed with PFA, permeabilized and labeled with DAPI to stain nuclei. Single confocal sections show Cherry-tagged protein fluorescence in red and DAPI staining in blue.

We finally tested if hPIV3-C_90-195_ was able, like full-length hPIV3-C, to block IFN-α/β signaling and enhance cellular response to EGF stimulation. First, cells were transfected with 3×FLAG-tagged hPIV3-C, hPIV3-C_90-195_ or hPIV3-C_1-89_ together with the IFN-α/β reporter plasmid, and stimulated 24 h later with recombinant IFN-β. Reporter gene expression was determined 24 h post transfection and found to be inhibited exclusively by full-length hPIV3-C (Figure 8A). Although this may reflect the weakness of hPIV3-C interaction with STAT1 (Figure 7B), this also indicates that both the N-terminal and C-terminal regions of hPIV3-C are required to block IFN-α/β signaling, even if only the C-terminal region is required for the binding to STAT1. The same constructs were tested using the Elk1 activity reporter plasmids (Figure 8B). Again, only full-length hPIV3-C was able to enhance Elk1 activation upon EGF stimulation whereas full-length hPIV3-C and hPIV3-C_90-195_ were expressed at similar levels in transfected cells (Figure 8B, upper right panel).

Because GRB2 binding to hPIV3-C and hPIV3-C_90-195_ were essentially equivalent in co-affinity purification experiments, we hypothesized that the N-terminal region of hPIV3-C was required for its proper subcellular localization. Thus, hPIV3-C, hPIV3-C_90-195_ and hPIV3-C_1-89_ were expressed in fusion downstream of the red fluorescent protein Cherry. As shown in Figure 8C, Cherry alone or fused to hPIV3-C_90-195_ localized both in the nucleus and the cytoplasm of transfected cells. In contrast, full-length hPIV3-C essentially accumulated at the cellular membrane whereas hPIV3-C_1-89_ was in the nucleus. Although we have no explanation for this unexpected localization of hPIV3-C_1-89_, these observations show that only full-length hPIV3-C is able to target the cellular membrane where both IFN-α/β and EGF signaling are triggered.

## Discussion

*Paramyxoviridae* have evolved various mechanisms to block IFN-α/β response, in particular signaling downstream IFNAR1/IFNAR2c receptor [20],[33]. Although members of *Pneumovirinae* subfamily have specific genes to encode inhibitors of IFN-α/β signaling pathway, those expressed by other *Paramyxoviridae* (*i.e. Paramyxovirinae* subfamily) are encoded by overlapping reading frames embedded within the gene P. *Rubulaviruses* express V proteins that target STAT1 and/or STAT2 for ubiquitination and degradation, while *Morbilliviruses* and *Henipaviruses* V proteins essentially impair STAT1/2 phosphorylation, activation and nuclear translocation. In addition, *Morbilliviruses* and *Henipaviruses* also encode for C proteins of which role in the inhibition of IFN-α/β response has been a matter of debates [34]–[37]. Recent reports showed that *Morbillivirus* C proteins only have a minor role in the inhibition of IFN-α/β signaling [37], but are essential to block IFN-α/β induction [38]. Whether *Henipavirus* C proteins can directly block IFN-α/β or promote viral replication through alternative mechanisms is unclear [35]. In contrast, it has been clearly established that *Respirovirus* C proteins are potent inhibitors of IFN-α/β signaling [5], [18], [39]–[41]. In this report, we show that hPIV3-C, but not MV-C or Nipah-C, directly interacts with STAT1 and efficiently inhibits IFN-α/β signaling. In addition, we identified a minimal STAT1 binding domain that encompasses AA 90-195 of hPIV3-C, a region suspected to fold into a coiled coil. Interestingly, this conserved domain is localized within the STAT1 binding region shared by all four isoforms of Sendai virus C protein [32]. Together, these results confirm the capacity of hPIV3-C to block IFN-α/β signaling pathway [18], provide molecular basis to this inhibition and clarify the fact that *Respirovirus* C proteins are functionally distinct from *Morbillivirus* and *Henipavirus* C proteins.

In addition to STAT1, we show that hPIV3-C interacts directly with GRB2 and enhances MAPK/ERK signaling downstream of EGF receptor (EGFR). Our data give the first example of a *Paramyxoviridae* protein that contributes to the stimulation of EGFR and MAPK/ERK pathway and provides molecular basis to this activity. This pathway has been known for decades as a prime target of DNA tumor viruses and oncogenic retroviruses, and its activation represents an essential step toward carcinogenesis [3],[42]. But recent data demonstrate that non-oncogenic RNA viruses also activate this signaling cascade to support viral replication and spreading [31]. Whether it is activated upon EGFR engagement or other means, MAPK/ERK pathway regulates a multiplicity of cellular processes including proliferation, differentiation, development, cell survival and inflammation. As a consequence, how the activation of MAPK/ERK pathway promotes viral replication is a complex question. Interestingly, two non-oncogenic RNA viruses associated with acute respiratory tract infections have been recently reported to modulate the EGFR pathway. Both human respiratory syncytial virus (hRSV), a member of *Paramyxoviridae* like hPIV3, and a rhinovirus that belongs to *Picornaviridae* family activate EGFR and MAPK/ERK pathway [25],[27]. Infection of epithelial cells by these viruses stimulates the processing and activation of EGFR ligands by membrane matrix metalloproteinase and subsequent engagement of EGFR through autocrine/paracrine mechanisms. Experiments performed on rhinovirus show that viral replication and TLR3 engagement by viral RNA are both required to activate the EGFR and MAPK/ERK pathway [27]. In this report, we show that hPIV3 infection also activates MAPK/ERK pathway in the absence of external stimuli, a phenomenon that possibly relies on the engagement of pathogen recognition receptors. Although hPIV3-C alone is unable to activate this pathway, our data suggest that expression of this virulence factor enhances MAPK/ERK activation above normal level in infected cells, thereby contributing to viral replication and pathogenesis.

Induction of MAPK/ERK pathway by RNA viruses has numerous consequences on cell biology. First, it results in increased expression of inflammatory factors, in particular cytokines and chemokines that recruit cellular effectors of immunity [27], [43]–[47]. MAPK/ERK pathway was also reported to block the antiviral response induced by IFN-α/β, making its activation beneficial to virus replication [29]. Another consequence of MAPK/ERK pathway activation is the induction of mucin production by infected epithelial cells [24],[27]. Although mucin expression is a critical innate defense system, excessive production of mucus results in the obstruction of airways and delays the elimination of pathogens. Finally, it has been demonstrated *in vitro* that upon hRSV infection, activation of EGFR and MAPK/ERK pathway sustains viral replication by retarding the death of infected cells [25]. Altogether, these data suggest that although a moderate activation of MAPK/ERK pathway contributes to the innate response against viruses, an excessive activation leads to deleterious inflammation, inhibition of IFN-α/β response, airway obstruction and infected cell survival [28]. Therefore, it is tempting to speculate that hPIV3-C interaction with GRB2 and EGFR pathway participates in such deregulation of airway epithelium homeostasis to promote hPIV3 replication and spreading. A consequence of such perturbations could be an aggravation of chronic inflammatory airway diseases like asthma or chronic obstructive pulmonary disease as already suggested by epidemiological links with *Paramyxoviridae* infections and *in vivo* models [48],[49].

Besides its effects on immune response, activation of MAPK/ERK pathway has direct consequences on viral replication as assessed by *in vitro* experiments. It is now well documented that MAPK/ERK pathway inhibition with U0126 or PD098059 deeply impairs the replication of numerous RNA viruses including hRSV ([50]; and for review see [31]). Similarly, we show in this report that MAPK/ERK pathway inhibition prevents hPIV3 protein expression in infected cells as assessed by hPIV3-HN detection. In influenza virus infected cells, membrane accumulation of influenza virus hemagglutinin (HA) induces lipid-rafts clustering that leads to MAPK/ERK pathway activation and nuclear export of viral ribonucleoprotein complexes to achieve viral particles assembly [51]–[53]. Because hPIV3 replication cycle is only cytoplasmic, mechanisms involved are necessarily distinct. A possible link between MAPK/ERK pathway and hPIV3 protein expression lies in the fact that among downstream targets of this pathway are essential factors of cellular translational machinery.

We show that hPIV3-C expression enhances the phosphorylation of S6 and eIF4E. The small ribosomal subunit protein S6 is the major phosphoprotein of eukaryotic ribosomes with five phosphorylation sites (Ser235, Ser236, Ser240, Ser244, and Ser247). Two families of serine/threonine kinases phosphorylate S6 *in vitro*: S6K1/2 and p90 ribosomal S6 kinase (RSK). Recently it has been shown that MAPK/ERK signaling pathway activates RSK family members that contribute to S6 phosphorylation on Ser235/236 thereby stimulating cap-dependent translation [23]. In addition, eIF4E that interacts with the cap structure and brings translation initiation factors together with the small ribosomal subunit *via* the scaffold protein eIF4G, undergoes regulated phosphorylation on Ser209 upon MAPK/ERK pathway activation. This phosphorylation event is dependent on eIF4G-associated MAPK signal-integrating kinases, Mnk1 and Mnk2 [22]. eIF4E is believed to be the least abundant of all initiation factors and therefore considered as a perfect target to regulate protein synthesis. Even though there is no direct link between eIF4E phosphorylation and the enhanced translation observed, the fraction of phosphorylated eIF4E dramatically increases following treatment of the cells with growth factors, hormones and mitogens. Therefore, eIF4E phosphorylation has been associated with increased translation rates. hPIV3 mRNAs are capped and polyadenylated like their host counterparts. Thus, S6 and eIF4E phosphorylation together with a high level of viral gene transcription may contribute to a rapid switch toward viral protein synthesis within infected cells.

Specific biochemical investigations are still required to decipher how hPIV3-C can both inhibit IFN-α/β signaling and enhance EGFR and MAPK/ERK pathway. When searching the literature for viral proteins that target GRB2, we found specific reports on NS5A from hepatitis C virus and ORF3 from hepatitis E virus [54],[55]. Although NS5A inhibits MAPK/ERK activation induced by exogenous EGF, ORF3 was described as an activator of MAPK/ERK pathway like hPIV3-C. Both NS5A and ORF3 exhibit a proline-rich motif (PXXP) to bind the Src homology 3 (SH3) domains of GRB2, but there is no such motif in hPIV3-C suggesting that other mechanisms mediate STAT1 and GRB2 binding. Interestingly, these two cellular proteins exhibit SH2 domains. Such domains typically bind a phosphorylated tyrosine residue in the context of a longer peptide motif within a target protein. Although there is no evidence that hPIV3-C becomes phosphorylated, we have tested hPIV3-C interaction with mutant STAT1 and GRB2 exhibiting SH2 domains disabled for the interaction with phosphotyrosine residues. These mutants were not affected for the interaction with hPIV3-C (data not shown). This suggests that hPIV3-C either binds distinct regions of STAT1 and GRB2, or interacts with a region of the SH2 domain that does not involve the phosphotyrosine binding site. Finally, our results also show that full-length hPIV3-C is required to modulate IFN-α/β and MAPK/ERK pathways since AA 90-195 that bind STAT1 and GRB2 are unable to do so when expressed alone. Full-length hPIV3-C was also required to observe a localization at the cell membrane, suggesting a link with its activity. Interestingly, the N-terminal 23 residues of Sendai virus C protein act as a membrane targeting signal [56]. But the N-terminal residues of hPIV3-C (AA 1-89) were unable to do so, and sequence analysis did not show any conservation with the C protein of Sendai virus. Thus, hPIV3-C tertiary structure is apparently required to target this protein at the cell membrane. This specific localization could both sequester STAT1 to prevent the stimulation of IFN-target genes and contribute to the aggregation of GRB2-SOS complexes to enhance MAPK/ERK signaling [57]. Altogether, this suggests that hPIV3-C interaction with STAT1 and GRB2 represents a potential target for the development of antiviral molecules against hPIV3 and possibly other members of *Respirovirus* genus.

## Materials and Methods

### Plasmid DNA constructs

P-encoding sequence from hPIV3 wild-type strain (DF042505) was amplified by RT-PCR (Titan One tube; Roche Applied Science) from total RNA purified from infected cells (RNeasy kit; Qiagen). Amplification was performed using the following hPIV3-P specific primers flanked with Gateway cloning sites: 5′-ggggacaactttgtacaaaaaagttggcatgGAAAGCGATGCTAAAAACTATCAAA and 5′-ggggacaactttgtacaagaaagttggttaTTGGCAATTATTGACATCTTCATTGAAC. PCR products were cloned using TOPO TA Cloning kit (Invitrogen) into TOPO vector. A total of 21 clones were analyzed to establish the sequence of hPIV3-P (GenBank ID: EU719627). Interestingly, 8 clones were not edited, 11 clones were edited by the addition of one G residue, and 2 clones were edited by the addition of 5 G residues. One of the plasmids containing the unedited sequence of hPIV3-P was selected and subsequently used as a template to clone hPIV3-C.

DNA sequences encoding full-length hPIV3-C or fragments corresponding to AA 1-89 or 90-195 were amplified by PCR from p(hPIV3-P)-TOPO and cloned by *in vitro* recombination into pDONR207 (Gateway system; Invitrogen) as previously described [58]. Similarly, MV-C was amplified from p(+)MV323 that contains the full genome of measles virus wild-type strain Ichinose (kindly provided by Dr. K. Takeuchi, [59]). Nipah-C was amplified from NiV-P plasmid (kindly provided by Dr. TF. Wild; [60]). GRB2 coding sequence was amplified from the human spleen cDNA library used to perform the yeast two-hybrid screen (Invitrogen). The pDONR207 plasmid containing STAT1 was previously described [58]. Viral or cellular coding sequences were subsequently transferred by *in vitro* recombination from pDONR207 into different Gateway-compatible destination vectors (see below) following manufacturer's recommendation (LR cloning reaction, Invitrogen). To perform yeast two-hybrid experiments, coding sequences were recombined into pPC86 (Invitrogen) to be expressed in fusion downstream of the activation domain of Gal4 (Gal4-AD) or into pDEST32 to be expressed in fusion downstream of the DNA binding domain of Gal4 (Gal4-DB). In mammalian cells, GST-tag and 3×FLAG-tag fusions were achieved using pDEST27 (Invitrogen) or pCI-neo-3×FLAG vector, respectively [61]. We also used pCI-neo (Promega) and pmCherry-C1 (Clontech) to express proteins without a tag or in fusion downstream of Cherry, respectively. These two plasmids were made Gateway-compatible using the Gateway vector conversion system (Invitrogen).

### Cell lines, viral infections and flow cytometry analysis

HEK-293T, Hela and Vero cells were maintained in Dulbecco's modified Eagle's medium (DMEM; Gibco-Invitrogen) containing 10% fetal bovine serum, penicillin, and streptomycin at 37°C and 5% CO_2_. A549 and BEAS-2B cells were maintained in F-12K medium (Gibco-Invitrogen) containing 10% fetal bovine serum, penicillin, and streptomycin at 37°C and 5% CO_2_. hPIV3 (strain C243) was amplified and titrated on Vero cells following recommendations of ATCC (American Type Culture Collection). Recombinant MV-EGFP virus used in Figure S2 has been previously described [62]. Infections were performed for 2 h at 37°C in Optimem (Gibco-Invitrogen). Later on, cells were washed and incubated in fresh culture medium for 24 or 48 h. To detect viral replication, cells were recovered and incubated in PBS-paraformaldehyde 3.2% for 20 min. After extensive washing with PBS, cells were permeabilized with PBS-Triton 0.05% for 15 min, and then incubated with a monoclonal antibody specific to hPIV3-HN (M02122321, Abcam). Cells were washed and incubated in the presence of an anti-mouse Cy3-conjugated antibody (Jackson Immunoresearch). After extensive washing, cellular immuno-staining was analyzed using a FACSCalibur flow cytometer (BD). When specified, cells were pre-treated with MEK1/2 specific inhibitor U0126 (20 µM final; Promega) for 2 h before, during and after infection to study the impact on hPIV3 infection.

### Co-affinity purification experiments

To perform co-affinity purification experiments, cloned ORFs were transferred from pDONR207 to pDEST27 expression vector (Invitrogen) to achieve GST fusion, and to pCI-neo-3×FLAG vector [61] for 3×FLAG-fusion. Cell transfections were performed using Lipofectamine 2000 (Invitrogen). Unless specified otherwise, 5×10^5^ HEK-293T cells were dispensed in each well of a 6-well plate, and transfected 24 h later with 600 ng of each plasmid DNA per well. Two days post transfection, HEK-293T cells were washed in PBS, then resuspended in lysis buffer (0.5% Nonidet P-40, 20 mM Tris–HCl at pH 8, 120 mM NaCl and 1 mM EDTA) supplemented with Complete Protease Inhibitor Cocktail (Roche). Cell lysates were incubated on ice for 20 min, and then clarified by centrifugation at 14,000×g for 10 min. For pull-down analysis, 400 µg of protein extracts were incubated for 1 h at 4°C with 25 µl of glutathione-sepharose beads (Amersham Biosciences) to purify GST-tagged proteins. Beads were then washed 3 times in ice-cold lysis buffer and proteins were recovered by boiling in denaturing loading buffer (Invitrogen).

### Western blot analysis

Purified complexes and protein extracts were resolved by SDS-polyacrylamide gel electrophoresis (SDS-PAGE) on 4–12% NuPAGE Bis–Tris gels with MOPS running buffer (Invitrogen), and transferred to a nitrocellulose membrane. Proteins were detected using standard immunoblotting techniques. 3×FLAG- and GST-tagged proteins were detected with a mouse monoclonal HRP-conjugated anti-3×FLAG antibody (M2; Sigma-Aldrich) and a rabbit polyclonal anti-GST antibody (Sigma-Aldrich), respectively. Specific antibodies were used to detect endogenous STAT1 (clone-1; BD Biosciences), GRB2 (clone-81; BD Biosciences), phospho-ERK1/2 (clone-12D4; Upstate), ERK1/2 (CT; Upstate), phospho-eIF4E (Ser209; Cell Signaling), eIF4E (Cell Signaling), phospho-S6_235-236_ (Ser235/236; Cell Signaling), S6 (clone-54D2; Cell Signaling) and hPIV3-HN (M02122321; Abcam). Secondary anti-mouse and anti-rabbit HRP-conjugated antibodies were from GE-Healthcare. Densitometric analysis of the gels was performed using a specific module of Photoshop CS3 Extended (Adobe Systems Inc.).

### Yeast two-hybrid screening and gap-repair procedure

Our yeast two-hybrid protocols have been described in details elsewhere [58]. Briefly, pDEST32 plasmid encoding Gal4-DB fused to hPIV3-C was transformed in AH109 yeast strain (Clontech), and used to screen by mating a human spleen cDNA library cloned in the Gal4-AD pPC86 vector (Invitrogen) and previously established in Y187 yeast strain (Clontech). Yeast cells were plated on a selective medium lacking histidine and supplemented with 10 mM 3-amino-triazole (3-AT; Sigma-Aldrich) to select for interaction-dependent transactivation of HIS3 reporter gene. AD-cDNAs from [His+] colonies were amplified by PCR and sequenced to identify the host proteins interacting with hPIV3-C.

The gap-repair procedure was used to map the minimal portion of hPIV3-C interacting with STAT1 and GRB2. As previously described [63], both forward and reverse PCR primers were designed along the sequence of hPIV3-C and fused to specific tails allowing yeast-based recombination in Gal4-DB two-hybrid vector. Matrix combinations of forward and reverse primers were used to amplify fragments of hPIV3-C by PCR. AH109 yeast cells expressing AD-fused STAT1 or GRB2 were co-transformed with 5 µL of each PCR product in the presence 50 ng of linearized pDEST32 vector to achieve recombinatorial cloning by gap-repair. Fragments of hPIV3-C fused to Gal4-DB were then tested for interaction with AD-STAT1 or AD-GRB2 by plating yeast cells on selective medium lacking histidine and supplemented with 10 mM of 3-AT.

### Luciferase reporter gene assay

HEK-293T, Hela or Vero cells were plated in 24-well plates (2×10^5^ cells per well). One day later, cells were transfected with either pISRE-Luc (0.3 µg/well; Stratagene) or pFA2-Elk1 (0.3 µg/well; Stratagene) and pGal4-UAS-Luc plasmids (0.3 µg/well; provided by Dr. Y. Jacob) together with pRL-CMV reference plasmid (0.03 µg/well; Promega). Cells were simultaneously co-transfected with 0.3 µg/well of pCI-neo-3×FLAG, pCI-neo or pmCherryC1 expression vectors encoding viral proteins as specified. 24 h after transfection, cells were stimulated with IFN-β (Biosource) at 1000 IU/ml or starved for 6 h then stimulated with EGF (Upstate) at 100 ng/ml. 24 h post transfection, cells were lysed, and both firefly and *Renilla* luciferase activities in the lysate were determined using the Dual-luciferase Reporter Assay System (Promega). Reporter activity was calculated as the ratio of firefly luciferase activity to reference *Renilla* luciferase activity, and normalized so that positive control activity equals 100. When indicated, cells were treated with U0126 (Promega) at 20 µM final concentration upon EGF stimulation.

### Subcellular localization of Cherry-tagged hPIV3-C_FL_, C_1-89_ and C_90-195_

24-well plates containing coverslips were seeded with HEK-293T cells (2×10^5^ cells per well). One day later, cells were transfected with pmCherryC1 expression vector alone or encoding hPIV3-C_FL_, hPIV3-C_1-89_ or hPIV3-C_90-195_. 36 h after transfection, cells were incubated with PBS-PFA 4% for 20 min at RT, then treated with PBS-Triton 0.05% for 15 min at RT to permeabilize the cells. Finally, cells were incubated for 10 min at RT in a PBS-PFA 4% solution containing DAPI (4′-6-Diamidino-2-phenylindole) at 10 µg/ml. Preparations were mounted using Fluoromount-G (Southernbiotech), and imaging performed using a SP5 confocal miscroscope (Leica).

## Supporting Information

Figure S1 ERK1/2 phosphorylation is enhanced by hPIV3 infection in A549 cells. A549 cells were infected with hPIV3 (MOI = 3) and after 24 h, cells were starved for 12 h before stimulation with 100 ng/ml of EGF. Phosphorylation of ERK1/2 was determined by western blot analysis at 10 min, 30 min and 2 h post stimulation. hPIV3 infection was confirmed by anti-hPIV3 hemagglutinin-neuraminidase (hPIV3-HN) immunoblotting.
(7.34 MB TIF)

Figure S2 MEK1/2 inhibitor U0126 has no effect on MV protein synthesis. HEK-293T cells were left untreated (A) or treated with 20 µM of U0126 for 2 h (B). Then, cells were mock-treated or infected with a recombinant MV strain expressing EGFP (MOI = 1) and cultured with or without U0126 (A and B, respectively). 48 h after infection, EGFP expression was quantified by flow cytometry analysis.
(7.34 MB TIF)
