# Supplementary figures and images for: Differential Regulation of Type I Interferon and Epidermal Growth Factor Pathways by a Human Respirovirus Virulence Factor
Source: PLoS Pathog. 2009 Sep 18;5(9):e1000587. doi: 10.1371/journal.ppat.1000587 (PMC2736567; doi:10.1371/journal.ppat.1000587)

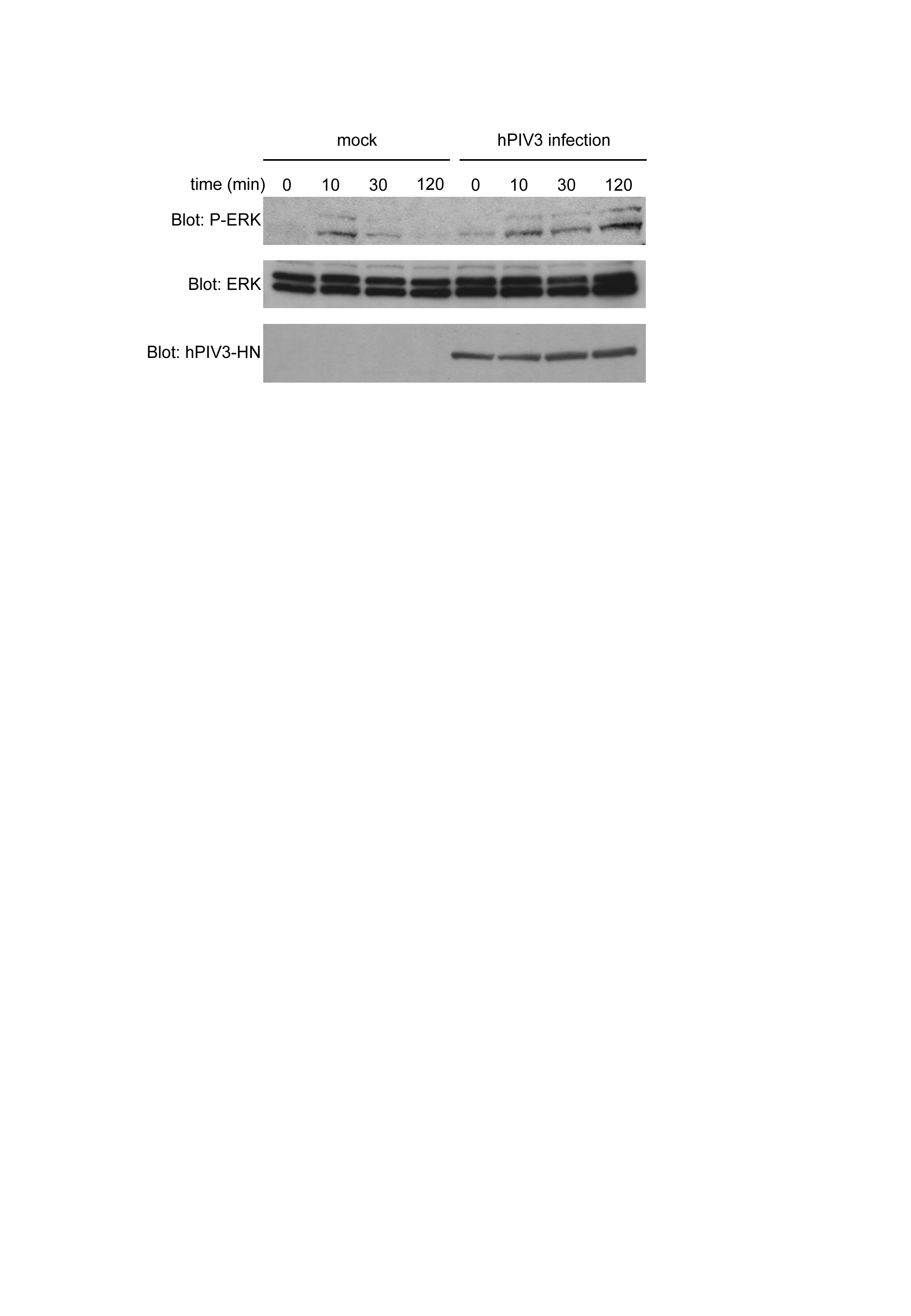

Supplement: Figure S1 — ERK1/2 phosphorylation is enhanced by hPIV3 infection in A549 cells. A549 cells were infected with hPIV3 (MOI = 3) and after 24 h, cells were starved for 12 h before stimulation with 100 ng/ml of EGF. Phosphorylation of ERK1/2 was determined by western blot analysis at 10 min, 30 min and 2 h post stimulation. hPIV3 infection was confirmed by anti-hPIV3 hemagglutinin-neuraminidase (hPIV3-HN) immunoblotting. (7.34 MB TIF) [file ppat.1000587.s001.tif]

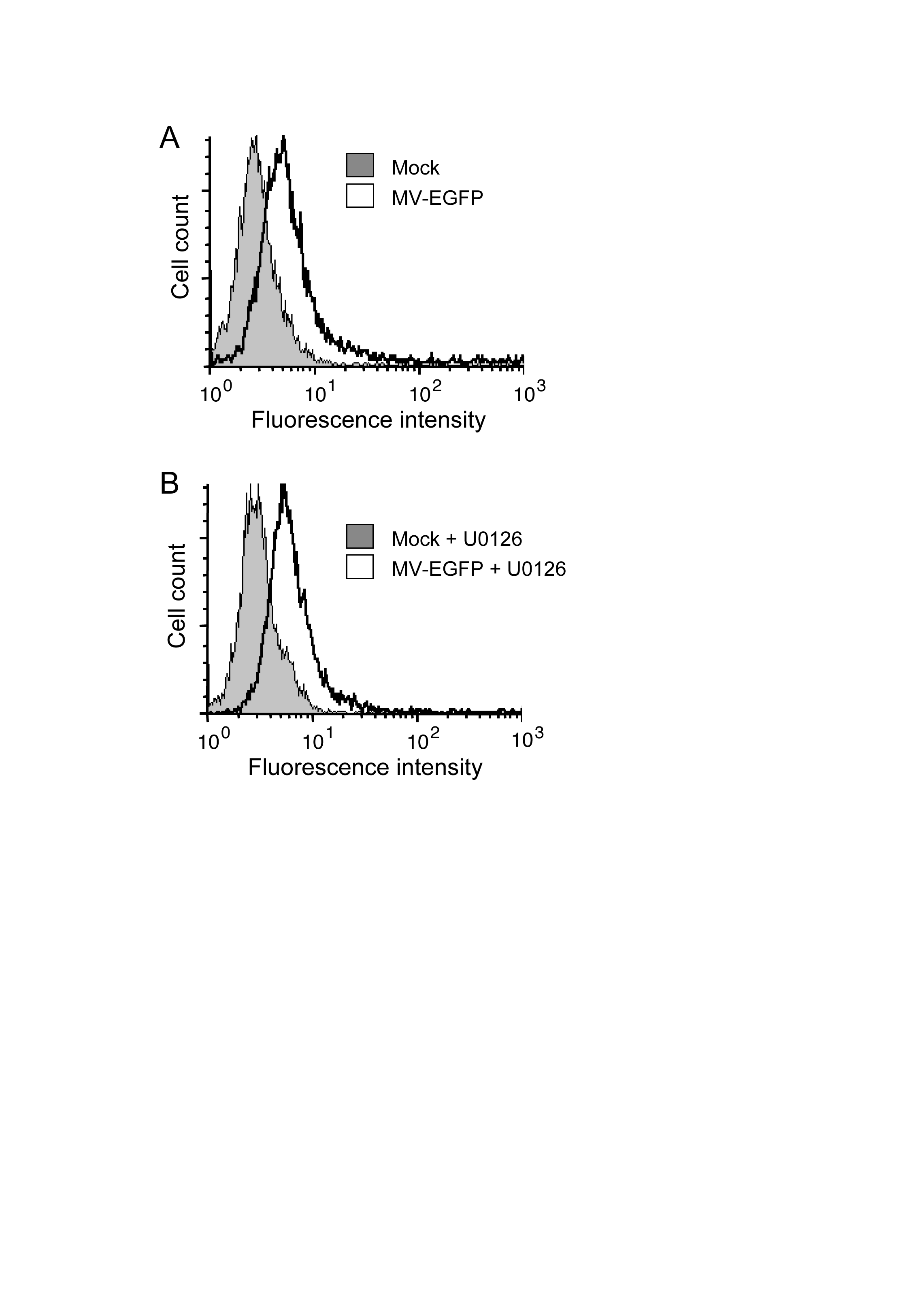

Supplement: Figure S2 — MEK1/2 inhibitor U0126 has no effect on MV protein synthesis. HEK-293T cells were left untreated (A) or treated with 20 µM of U0126 for 2 h (B). Then, cells were mock-treated or infected with a recombinant MV strain expressing EGFP (MOI = 1) and cultured with or without U0126 (A and B, respectively). 48 h after infection, EGFP expression was quantified by flow cytometry analysis. (7.34 MB TIF) [file ppat.1000587.s002.tif]
